# Supplementary material for: Predicting severe stunting and its determinants among under-five in Eastern African Countries: A machine learning algorithms
Source: PLoS One. 2026 Jan 2;21(1):e0340221. doi: 10.1371/journal.pone.0340221 (PMC12758688; doi:10.1371/journal.pone.0340221)
Supplement: S1 File — (DOCX) [file pone.0340221.s001.docx]

# **Supplementary files**

**I. Supplementary Tables**

**Table 1.** Sample size of each country for the prediction of severe stunting Eastern African countries, 2012-2022.

| **No** | **Country** | **Survey year** | **Sample size** |
| --- | --- | --- | --- |
| 1 | Comoros | 2012 | 2387 |
| 2 | Zimbabwe | 2015 | 4957 |
| 3 | Ethiopia | 2016 | 8855 |
| 4 | Uganda | 2016 | 4423 |
| 5 | Malawi | 2016 | 5149 |
| 6 | Burundi | 2016 | 6048 |
| 7 | Zambia | 2018 | 8746 |
| 8 | Rwanda | 2019 | 3809 |
| 9 | Madagascar | 2021 | 5778 |
| 10 | Tanzania | 2022 | 4807 |
| 11 | Mozambique | 2022 | 3733 |
| 12 | Kenya | 2022 | 17,324 |

**Table 2.** Performance comparison of the imputation techniques for the prediction of severe stunting Eastern African countries, 2012-2022.

| **Methods** | **Samples** | **Accuracy** | **F1 score** |
| --- | --- | --- | --- |
| Complete-case analysis | 35,103 | 0.886051 | 0.864268 |
| Mean/Mode Imputation | 76,019 | 0.895905 | 0.879402 |
| **KNN Imputation** | 76,019 | **0.897088** | **0.880405** |
| MICE Imputation | 76,019 | 0.896562 | 0.880321 |

**Hint:** KNN: K-Nearest Neighbors, MICE: Multiple Imputation by Chained Equations

**II. Supplementary Figures**

**
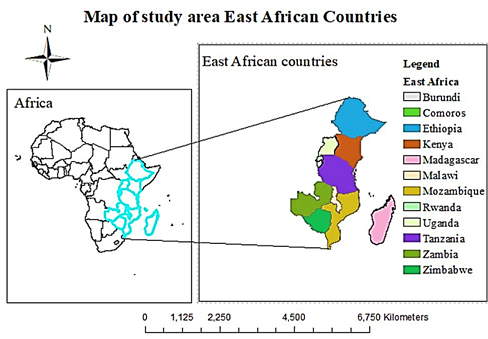
**

**Fig 1**. Map of study area (Eastern African Countries) for the predictions of severe stunting among under five, using DHS 2012-2012.


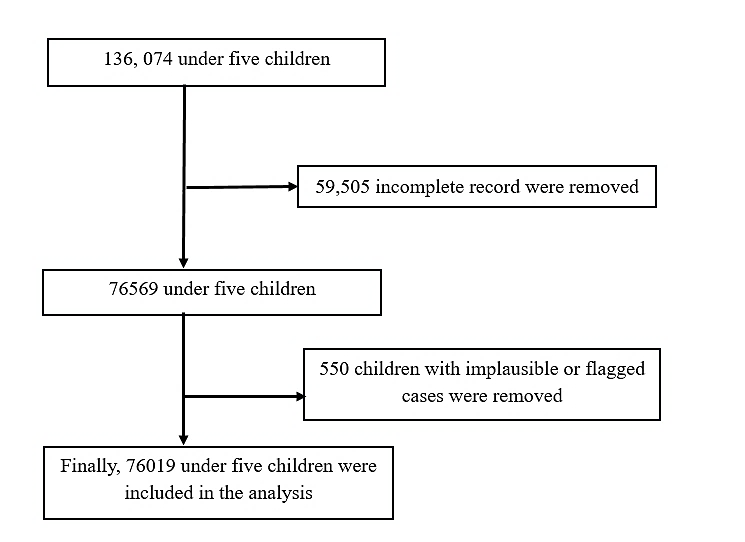


**Fig 2**. Sampling flow chart for the selection of the study participants in Eastern African countries, 2012-2022.


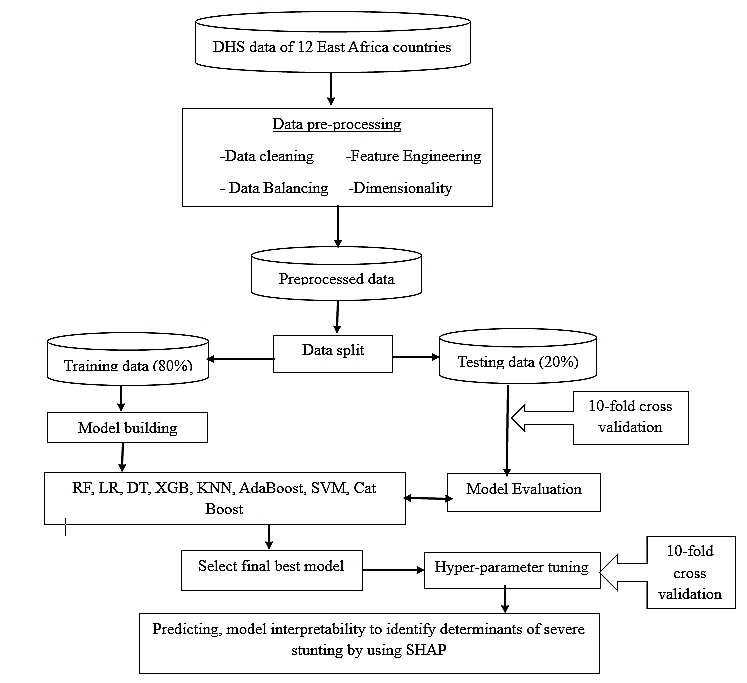


**Fig 3**. Workflow diagram to conduct the study for the prediction of severe stunting in Eastern African countries, 2012-2022.


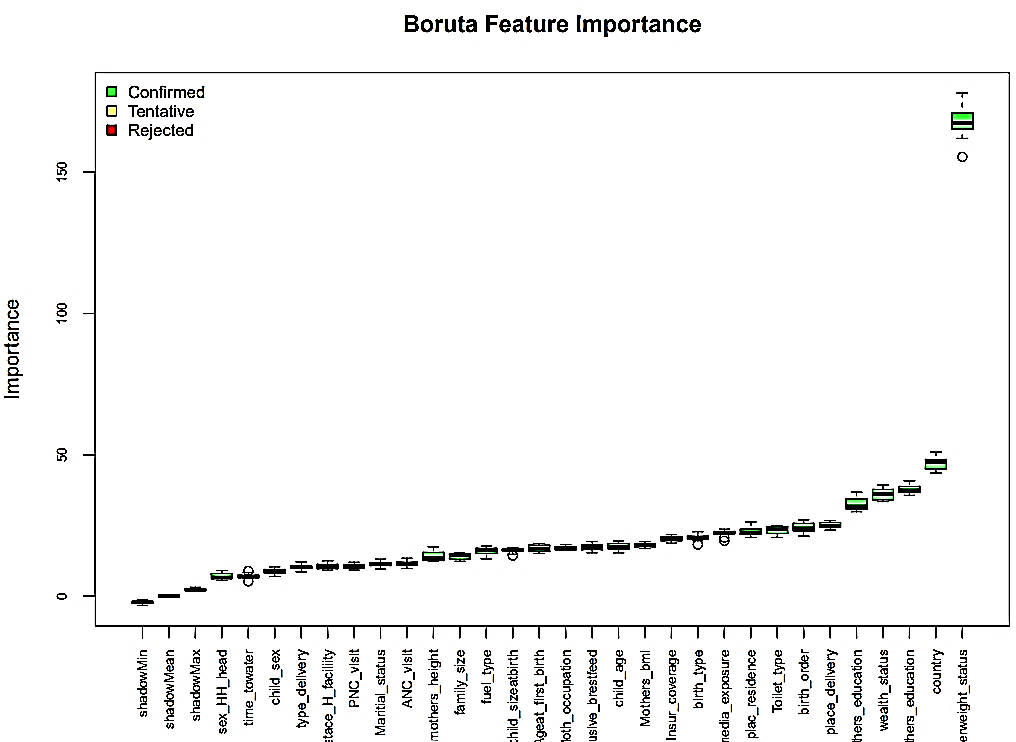


**Fig 4**. Boruta feature importance plot for the prediction of severe stunting Eastern African countries, 2012-2022.


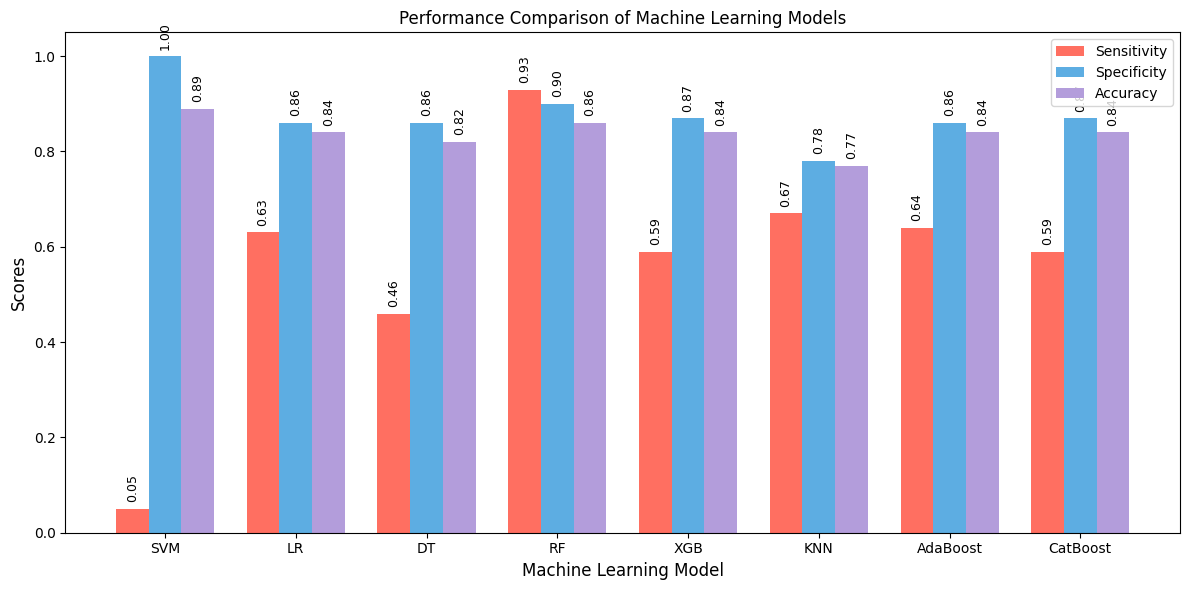


**Fig 5**. Performance evaluation of each models for the predictions of severe stunting among under five, in Eastern Africa countries 2012-2022*.*

*
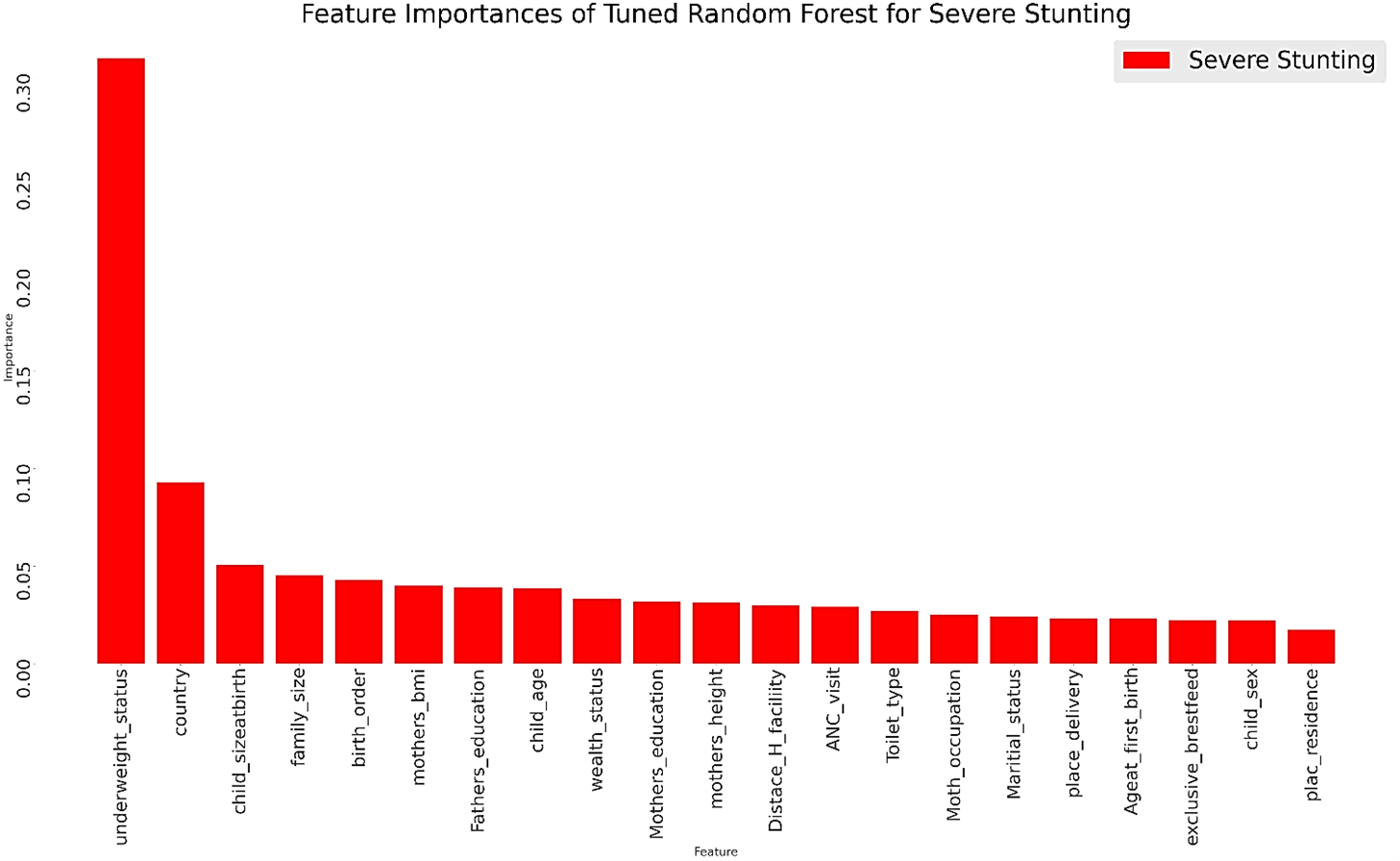
*

**Fig 6**. Feature importance for optimized random forest model for the predictions of severe stunting among under five, in Eastern Africa countries 2012-2022.


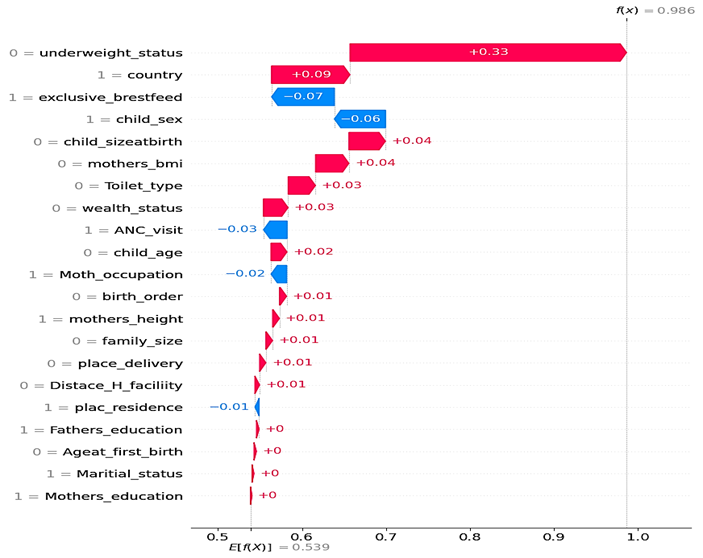


**Fig 7**. Local SHAP interpretation using waterfall plot, displaying the prediction for the first positive observation among under five, in Eastern Africa countries 2012-2022.

*Local SHAP interpretation include waterfall plot used to explain positive prediction, the waterfall plot begins with the expected value of model output on the x axis (E[f(x)]=0.539), which represents the initial prediction for the given sample before considering any feature contribution. These prediction is called initial prediction most common for the dataset calculated in the training sets serve as a reference point for feature contribution for a given observation if the model output above (E[f(x)]=0.539) it corresponds to a positive class(severe stunted) where as if it scores below (E[f(x)]=0.539) it corresponds to a negative class(not severely stunted). The combination of positive contributions (in red) and the negative contribution (blue) move the expected value output to the final prediction of total f(x)= 0.986 and classified as positive class(severely stunted) since it is greater than the base value (E[f(x)]=0.539).*


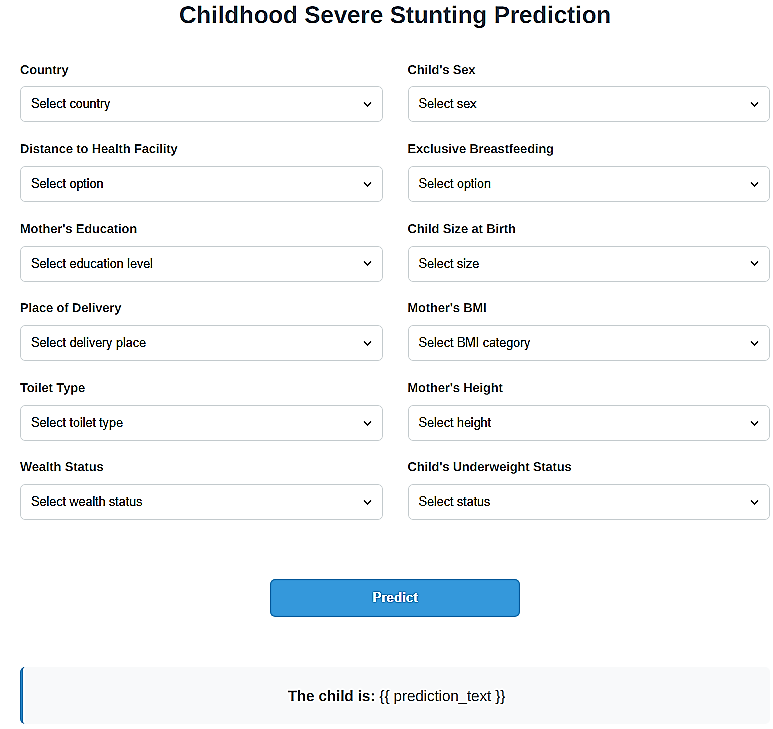


**Fig 8**: User interface for the deployed model to predict severe stunting among under five, in Eastern Africa countries 2012-2022.
